# Temperature-dependent coliphage induces distinct temporal bacterial morphological dynamics during infection

**DOI:** 10.1128/spectrum.04159-25

**Published:** 2026-05-27

**Authors:** Jiranan Pattano, Filosofia F. T. A. Prasasti, Songphon Buddhasiri, Patiphan Khunti, Panupon Mongkolkarvin, Parameth Thiennimitr, Poochit Nonejuie, Vorrapon Chaikeeratisak

**Affiliations:** 1 Department of Biochemistry, Faculty of Science, Chulalongkorn University 133942, Bangkok, Thailand; 2 Center for Advanced Therapeutics, Institute of Molecular Biosciences, Mahidol University 98841, Nakhon Pathom, Thailand; 3 Veterinary Public Health and Food Safety Centre for Asia Pacific, Faculty of Veterinary Medicine, Chiang Mai University 65101 , Chiang Mai, Thailand; 4 Department of Microbiology, Faculty of Medicine, Chiang Mai University 37686 , Chiang Mai, Thailand; 5 Center of Excellence in Microbial Diversity and Sustainable Utilization, Chiang Mai University 26682, Chiang Mai, Thailand; Universidad Nacional Autonoma de Mexico-Campus Morelos, Cuernavaca, Mexico

**Keywords:** bacterial cytological profiling, phage-induced morphological changes, bacteriophages, single cell analysis

## Abstract

Phage infection undergoes a series of physiological transitions, holding crucial information about phage replication dynamics and potential phage-derived antimicrobials. Although phage-induced cytological changes have been used to infer phage-hijacking mechanisms, current approaches are limited by the lack of comprehensive single-cell morphological analysis and insufficient resolution of temporal dynamics, particularly for phages displaying short latent periods, thereby hindering systematic characterization of morphological transitions throughout the infection cycle. Here, we characterized a newly identified coliphage with a genome of 53 kbp, Tiny, which exhibits an unusually long latent period, making it an ideal candidate for resolving temporal morphological transitions. Tiny exhibits both temperature- and host-dependent killing profiles against diverse *Escherichia coli* strains, including ATCC 25922, uropathogenic *E. coli*, and avian pathogenic *E. coli*. While its replication efficiency is temperature-dependent, showing enhanced productivity at lower temperatures, the duration of its adsorption and latent period is largely host-dependent. Depending on the bacterial strain, Tiny exhibits either a prolonged latent period in slow-adsorbing strains or a rapid one in fast-adsorbing strains, regardless of infection temperature, suggesting phage-host compatibility. Single-cell bacterial cytological profiling of Tiny-infected ATCC 25922 cells revealed that Tiny progressively induces distinct bacterial morphological transitions throughout its lytic cycle, suggesting sequential interference with host physiology as part of its replication cycle prior to cell lysis. This work establishes a broadly applicable framework for dissecting lytic phage biology with high temporal resolution and lays the foundation for future integrative omics studies aimed at understanding how phages sequentially modulate their bacterial hosts.

**IMPORTANCE:**

Antibiotics trigger unique patterns of morphological changes in bacteria, and these compound-specific signatures provide a basis for determining mechanisms of action in antibiotic discovery. By the same concept, phage-induced morphological changes can reveal key insights into phage replication dynamics and guide the identification of phage-derived antimicrobials. However, the complexity of phage biology and the variability of phage-host interactions pose challenges in interpreting these phenotypic outcomes. Here, we employed a phage-host pair that exhibits an unusually prolonged latent duration as a model to establish a broadly applicable framework for dissecting lytic phage biology with high temporal resolution. Through single-cell bacterial morphological analyses, this approach captures dynamic infection processes inducing morphological transitions across the phage replication cycle. This work provides a phenotypic analysis pipeline to advance our understanding of phage-host interactions and lays the foundation for future integrative omics studies to elucidate how phages sequentially modulate their bacterial hosts.

## INTRODUCTION

Over the next 25 years, global deaths attributable to multidrug-resistant (MDR) bacteria are projected to reach up to 39 million (1), representing a substantial increase compared to earlier estimates of approximately 10 million deaths in the absence of novel antibacterial drug development (2). This growing mortality estimate clearly signals that MDR infections will affect future generations, particularly large adult populations transitioning into older age, who represent the highest-risk group of MDR-associated mortality (1). Even though alternative antibacterial agents and approaches have been developed to combat MDR bacteria, significant challenges remain due to difficulties in their clinical implementation and the rapid emergence of MDR strains (1, 3). This issue underscores the urgent need to develop effective solutions, especially for bacterial groups classified by the World Health Organization (WHO) as critical-priority pathogens (4), including *Acinetobacter baumannii*, *Klebsiella pneumoniae*, *Mycobacterium tuberculosis*, *Enterobacter* spp., and *Escherichia coli*. Among these, MDR *E. coli* has emerged as a particularly severe threat, as it was ranked among the top 10 deadliest MDR pathogens worldwide in 2021 (5) and continues to contribute substantially to the global antimicrobial resistance crisis. Numerous surveillance studies have consistently reported that *E. coli* is one of the most antibiotic-resistant pathogens encountered in both hospital settings and livestock production systems (1, 6), thereby exacerbating the clinical and agricultural burden by severely restricting available treatment options.

MDR *E. coli* has become a growing global concern due to its ability to disseminate across humans, animals, and livestock production systems. Among its diverse pathotypes, extra-intestinal pathogenic *E. coli* (ExPEC), a zoonotic pathotype capable of causing disease in both mammals and birds, is of particular concern due to its high rates of antibiotic resistance. Several ExPEC lineages have been documented to circulate between animal and human populations, underscoring their significance as a public health threat (7). Within this group, uropathogenic *E. coli* (UPEC) frequently exhibits resistance to critical last-line antibiotics, such as carbapenems, colistin, and third-generation cephalosporins used in clinical settings (6, 8). Alarming levels of resistance have also been documented in UPEC strains isolated from companion animals, including cats and dogs, where strains show reduced susceptibility to commonly used antibiotics in veterinary hospitals (9, 10). In livestock farming, particularly poultry production, the long-term and extensive misuse of antibiotics for treatment, prophylaxis, and growth promotion has further driven the emergence of resistant lineages, including avian pathogenic *E. coli* (APEC), a predominant bacterial pathogen in chickens (11). A recent report by Ahmed et al. (12) further reinforced these concerns by showing a case in which MDR *E. coli* isolated from dead chickens on a commercial farm was subsequently transmitted to a farm worker, demonstrating direct zoonotic transfer. These reports emphasize that MDR *E. coli* is widely distributed, spreading extensively from its environmental and agricultural reservoirs to both animals and humans. This growing prevalence not only compromises the efficacy of available antibiotics but also underscores the urgent need for alternative strategies to mitigate the expanding threat posed by MDR *E. coli*.

Since then, many strategies have been developed to counter MDR bacteria, and several have progressed beyond laboratory concepts to real-world applications across food biocontrol, agriculture, and clinical practice. According to the latest WHO report (4), the global clinical development pipeline currently includes 90 antibacterial agents, comprising 50 traditional antibiotics and 40 alternative therapeutics. Within the category of alternative therapeutics, the WHO recognizes several types of agents, including antibodies, anti-virulence compounds, bacteriophages and phage-derived enzymes, immunomodulators, and microbiome-modulating agents. Particularly, bacteriophages are of particular interest. Phages eliminate their hosts through a highly specific killing cycle, consisting of four steps: attachment, genome replication, virion assembly, and host-cell lysis (13). Both phages and phage-encoded proteins are considered strong therapeutic candidates. The WHO has now included them within the clinical development pipeline, with 13 phage-based products currently under clinical evaluation, only 2 of which target MDR *E. coli* (4). Furthermore, many studies have demonstrated that whole-phage therapy displays robust efficacy against *E. coli* infection in animal models while minimizing disruption to gut microbiota composition (14, 15). This suggests that phage-based interventions have moved beyond *in vitro* studies and are now recognized as an effective strategy for controlling MDR *E. coli* in both clinical and livestock settings.

Even though phage applications have proven highly effective and are now widely implemented in many sectors, key limitations remain, particularly their narrow host specificity and the rapid emergence of phage-resistant bacteria when single phages are used (16). Despite these limitations, interest in phage-based technologies has not diminished. Instead, many researchers have shifted toward deeper investigations of phage-host interactions, with growing emphasis on phage-encoded degradative proteins that selectively target and disrupt bacterial cell envelopes. Among these proteins, endolysins are the most extensively studied and considered effective as they function to degrade the bacterial cell wall (17). Engineered endolysins and other phage-derived proteins have demonstrated broad-spectrum antibacterial activities (18, 19), and several studies have reported their safe use in preclinical settings (20). Despite this promising progress, the discovery of additional phage-derived antimicrobials remains challenging, largely due to the limited availability of high-quality reference genomes required for accurate functional annotation. As a result, phage genomes continue to represent an expansive yet underexplored reservoir of potentially novel antimicrobial agents.

Bacterial cytological profiling (BCP), a fluorescence microscopy-based platform for quantitative morphological analysis, has been widely used to elucidate mechanisms of action (MOAs) in diverse bacterial models, including *E. coli*, *Bacillus subtilis*, *Staphylococcus aureus*, *Vibrio parahaemolyticus*, and *Acinetobacter baumannii* (21–26). Based on the signature morphological changes induced by defined antibiotic MOAs, BCP can identify numerous antimicrobial molecules (24, 26–29) as well as those derived from bacteriophages (30). However, phage-induced morphological changes are relatively more complex than the phenotypes elicited by antibiotics, as phages employ diverse and multiple stage-specific host-hijacking strategies across a rapid infection period, particularly in compact-genome phages. Consequently, previous single-cell analyses, which were conducted manually and focused on a limited set of phenotypes (30), have provided only a partial view of the rich morphological information embedded within single-cell data sets. Here, we identified a novel coliphage, Tiny, classified within the genus *Carltongylesvirus*. Phage Tiny exhibits temperature-dependent infectivity and is capable of lysing various *E. coli* strains, including laboratory, uropathogenic, and avian pathogenic strains. Even though its infectivity is enhanced at 30°C, the duration of its lytic life cycle remains unaffected by temperature. Notably, Tiny displays a distinct prolonged latent period, reaching approximately 80 min, with a low adsorption rate and delayed killing activity in many *E. coli* strains, particularly ATCC 25922. This feature makes the Tiny-ATCC 25922 system particularly well suited for high-resolution BCP analysis aimed at capturing morphological transitions at finer resolution within the latent period. For the first time, we successfully reveal the temporal sequence of phage-induced morphological changes at the single-cell level throughout the entire lytic life cycle, offering insights into phage-host interactions associated with distinct bacterial morphological dynamics.

## RESULTS

### Temperature-dependent interactions between myophage Tiny and its hosts

**Fig 1:**
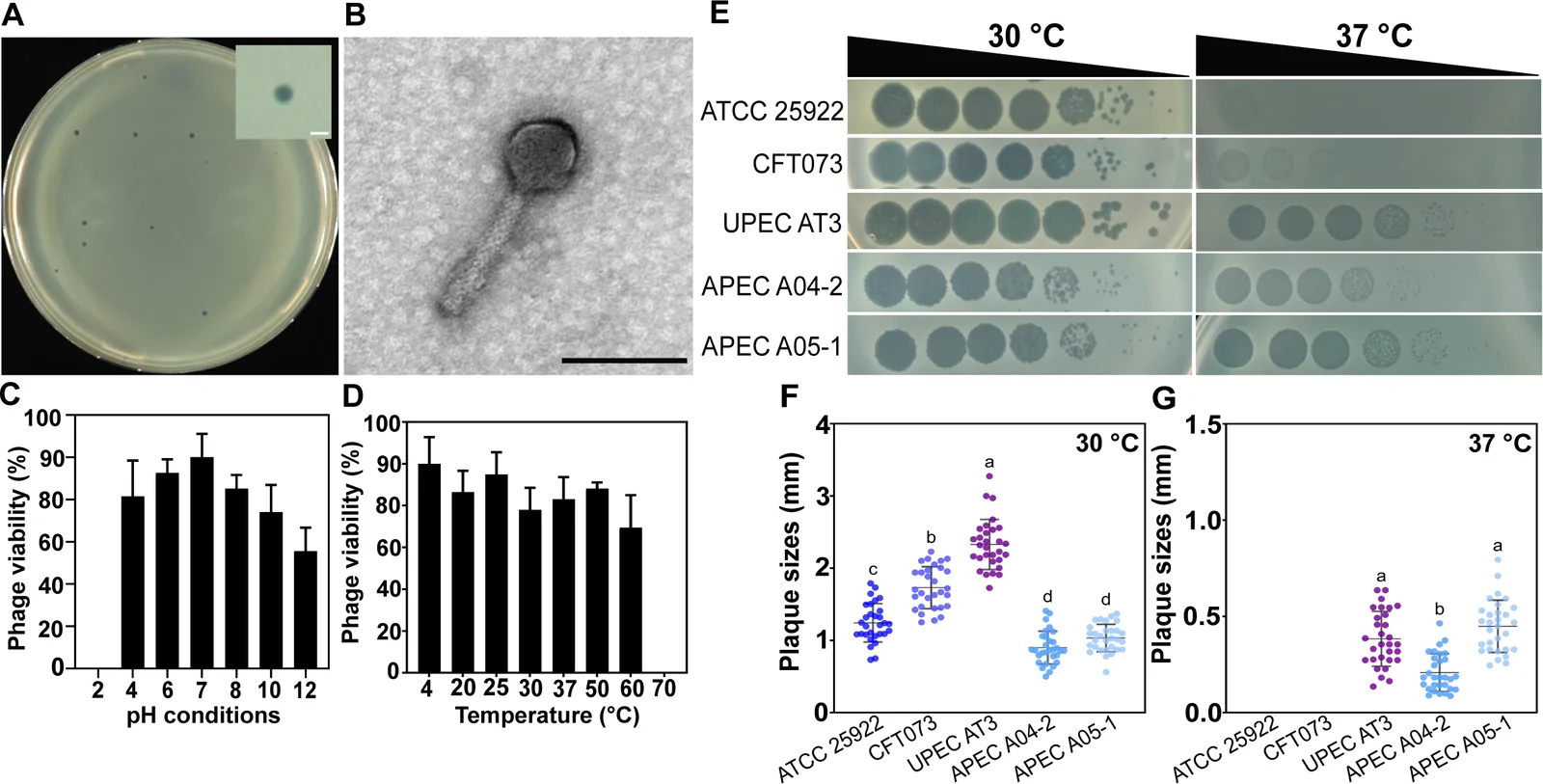
Morphological and biological characteristics of phage Tiny. (**A**) Plaque morphology of phage Tiny on a lawn of *E. coli* ATCC 25922. A single plaque (top right of panel A) is magnified. Scale bar equals 2 mm. (**B**) The structure of phage Tiny was visualized by a TEM. Scale bar equals 100 nm. (**C and D**) Viability of phage Tiny under different simulated environmental conditions: (**C**) pH and (**D**) temperature. (**E–G**) Influence of temperature on the infectivity and plaque morphology of phage Tiny across bacterial hosts. (**E**) The efficiency of plating (EOP) of phage Tiny against ATCC 25922, CFT073, UPEC AT3, APEC A04-2, and APEC A05-1 at 30°C and 37°C. (**F and G**) Plaque sizes formed on double-layer agar (DLA) lawns of *E. coli* strains at (**F**) 30°C and (**G**) 37°C. All data represent the mean ± standard deviation of at least three independent biological replicates. Multiple comparisons among bacterial strains were performed using one-way ANOVA with Tukey’s HSD *post hoc* test (*P* ≤ 0.05, *n* = 30). Different lowercase letters denote statistically significant differences, whereas the same letter represents no significant differences.

A library of coliphages targeting diverse *Escherichia coli* isolates was created as previously described (31). Among the coliphages in our library, one newly isolated phage, named “Tiny,” was able to efficiently propagate in *E. coli* ATCC 25922 used as a laboratory host strain, producing clear plaques with sharp, well-defined edges (Fig. 1A). When incubated at 30°C, this phage generated clear plaques with an average diameter of 1.24 ± 0.25 mm (*n* = 30) (Fig. 1A and E), whereas no plaques were detectable at 37°C (Fig. 1E). Based on transmission electron microscope (TEM) observations, the phage exhibited a myovirus-like morphology (Fig. 1B) according to the criteria defined by the International Committee on Taxonomy of Viruses (ICTV) (32), possessing an icosahedral capsid (height: 63.4 ± 9.7 nm, *n* = 10; width: 63.8 ± 7.4 nm, *n* = 10) and a contractile tail (length: 157.4 ± 17.4 nm, *n* = 10).

Phage Tiny tolerated a wide pH range (4–12) but completely lost infectivity at pH 2 (Fig. 1C). It also exhibited thermal stability across a broad temperature range, maintaining more than 60% viability after 1 h of incubation at 4°C–60°C (Fig. 1D), but becoming fully inactivated at 70°C. Despite this relatively robust thermal stability profile, the infectivity of Tiny was markedly temperature-dependent. While the phage effectively produced high titers of progeny in *E. coli* ATCC 25922 at 30°C, its infectivity dropped sharply at 37°C, resulting in an efficiency of plating (EOP) below 0.001, which is considered inefficient (Table 1; Fig. 1E).

**TABLE 1:** Host range determination and EOP of phage Tiny

Bacterial strains	Sources	EOP values^*a*^	
		30°C	37°C
*E. coli* ATCC 25922	American Type Culture Collection (serotype O6:H1)	1.000	≤0.001
*E. coli* MC4100	American Type Culture Collection	–	–
*E. coli* Nisslle 1917	Non-pathogenic probiotic strain (serotype O6:K5:H1)	–	–
UPEC (*n* = 9)			
*E. coli* CFT037	American Type Culture Collection (serotype O6:K2:H1)	0.958	0.005
*E. coli* UTI89	Symptomatic strain (serotype O18:K1:H7)	–	–
*E. coli* ABU83972	Asymptomatic strain (serotype O nt/K5)	–	–
AT1	Clinical strain (31)	–	–
AT2	Clinical strain (31)	–	–
AT3	Clinical strain (31)	0.625	0.792
AT4	Clinical strain (31)	–	–
AT5	Clinical strain (31)	–	–
AT6	Clinical strain (31)	–	–
APEC (*n* = 15)^*b*^			
DO1A	Highly virulent pathogenic strain	–	–
DO7A	Highly virulent pathogenic strain	≤0.001	≤0.001
D31D	Highly virulent pathogenic strain	–	–
D511A	Highly virulent pathogenic strain	–	–
D713A	Highly virulent pathogenic strain	–	–
D4416D	Highly virulent pathogenic strain	–	–
A04-2	Highly virulent pathogenic strain	0.923	0.352
A05-1	Highly virulent pathogenic strain	1.154	0.407
A11-1	Highly virulent pathogenic strain	–	–
A15-1	Highly virulent pathogenic strain	–	–
B01-1	Highly virulent pathogenic strain	–	–
B09-4	Highly virulent pathogenic strain	–	–
E02-5	Highly virulent pathogenic strain	–	–
E05-2	Highly virulent pathogenic strain	–	–
F01-5	Highly virulent pathogenic strain	–	–

^*a*^ EOP of phage Tiny against *E. coli* strains was conducted at 30°C and 37°C. EOP values were divided into four classifications: highly productive (≥0.5), intermediate productive (0.1 ≤ EOP < 0.5), low productive (0.001 < EOP < 0.1), and inefficient (≤ 0.001). No lysis plaques were interpreted as phage Tiny showing no killing capacity against *E. coli* strains and are represented as “–”.

^*b*^ APEC strains carried five virulence-associated genes (*iroN*, *ompT*, *hlyF*, *iss*, and *iutA*) in their genome, exhibiting highly virulent. Antimicrobial susceptibility profiles of 15 APEC strains are presented in Table S1.

Given the temperature-dependent infectivity observed in the parental host *E. coli* ATCC 25922, to further characterize the interaction of myophage Tiny across diverse *E. coli* isolates, we performed a host range assay and EOP analysis at two temperatures, 30°C and 37°C. The results showed that phage Tiny could infect 6 out of 27 *E. coli* strains, including the laboratory strain ATCC 25922 (parental strain), 2 UPEC isolates (31), and 3 APEC strains (Table 1; Table S1). An EOP assay was then performed to classify the infection efficiency of phage Tiny against these susceptible strains. The data demonstrated that phage Tiny tends to exhibit higher infectivity at 30°C than at 37°C (Table 1; Fig. 1E). At 30°C, Tiny showed strong infectivity with EOP above 0.5 toward five *E. coli* strains, including ATCC 25922, CFT073, UPEC AT3, APEC A04-2, and APEC A05-1. However, at 37°C, consistent with its loss of infectivity toward ATCC 25922 mentioned above, phage Tiny also showed markedly reduced infectivity against CFT073 (Table 1). Tiny still maintained immediate infectivity at 37°C in only two strains, APEC A04-2 and APEC A05-1. Notably, although the EOP of Tiny against UPEC AT3 slightly increased at 37°C, the plaque size was substantially reduced, suggesting a distinct phage-host interaction in this pair (Table 1; Fig. 1E through G). Together, these findings highlight the temperature- and host-specific factors that define the environmental conditions in which the phage can maintain strong infectivity and biological activity, with 30°C identified as the optimal condition.

### Phage Tiny is a newly isolated coliphage in the genus *Carltongylesvirus*

**Fig 2:**
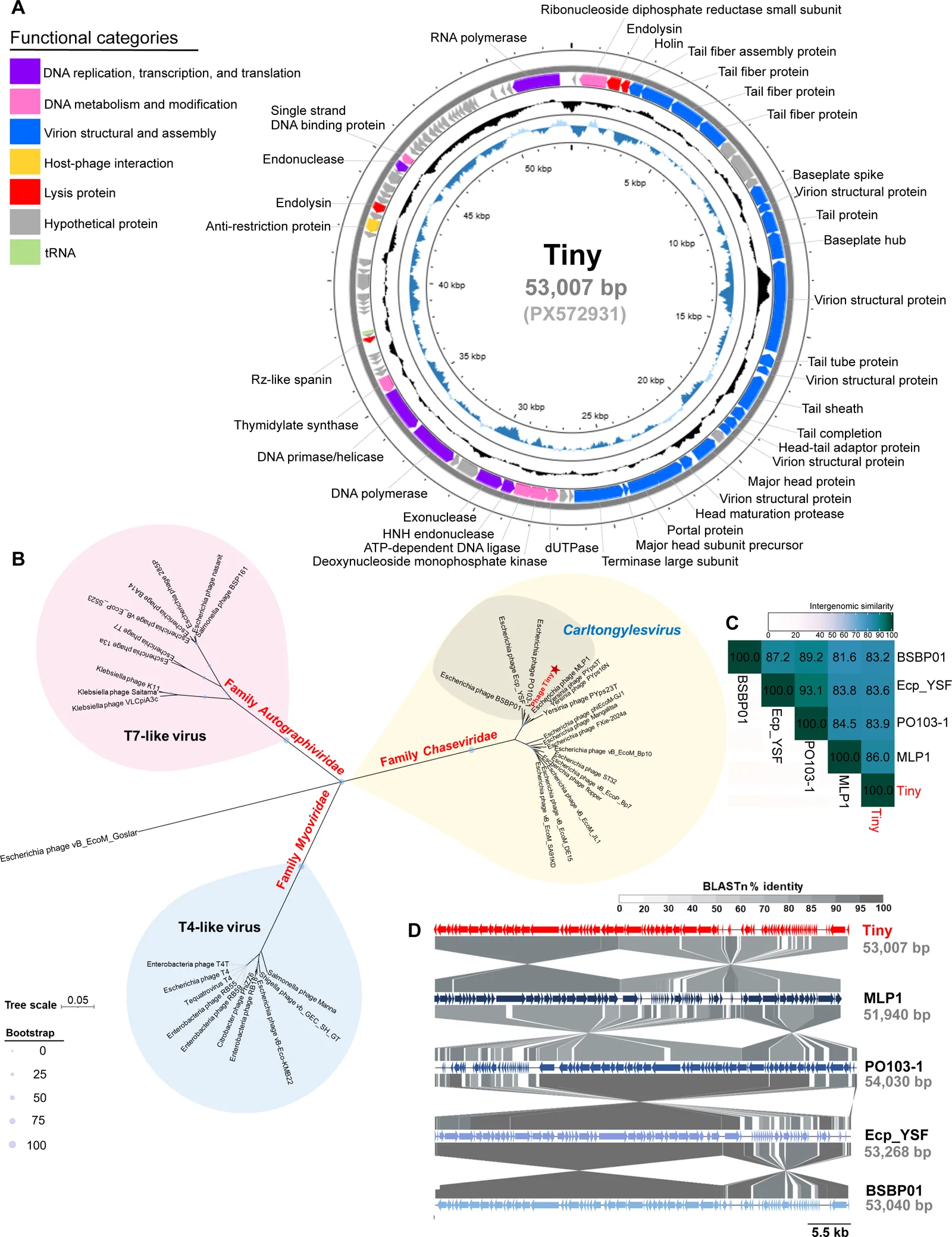
Phage Tiny is classified as a new member of the genus *Carltongylesvirus*, family *Chaseviridae*. (**A**) Genome visualization of phage Tiny (NCBI accession number: PX572931). The circular genome map illustrates the size (inner scale), GC skew (positive in blue and negative in light blue), and GC content (black). The predicted functions of ORFs are classified into seven categories as follows: DNA replication, transcription, and translation (purple); DNA metabolism and modification (pink); virion structure and assembly (blue); host-phage interaction (yellow); lysis proteins (red); hypothetical proteins (gray); and tRNAs (green). The ORFs directions are indicated by arrows in the outermost circular map. (**B**) Evolutionary phylogenetic tree of phage Tiny. The tree was constructed based on the complete genome of phage Tiny (red star) together with other 17 ICTV-representative members of the genus *Carltongylesvirus* (yellow area) and related *E. coli* phage families, including T7-like viruses (pink area) and T4-like viruses (blue area), using VICTOR with 100 bootstrap replicates. The nucleus-forming jumbophage Goslar genome was used as an outgroup. The tree was visualized with iTOL. Bootstrap values are represented by the size of the blue circles on the phylogenetic tree. The closest genetically related *E. coli* phages (MLP1, PO103-1, Ecp_YSF, and BSBP01) to Tiny are highlighted by a gray circle and were selected for comparative genomic analysis in panels **C** and **D**. (**C**) VIRIDIC heatmap showing intergenomic similarity between phage Tiny and four closely related *E. coli* phages. (**D**) Comparative genomic map of phage Tiny and four related phages, with arrows indicating ORF direction and gray-shaded lines representing homology levels.

Phage Tiny is a newly characterized *Escherichia* phage whose genome has been sequenced and analyzed to explore its functional features and assess its suitability for application. Tiny has a compact genome comprising 53,007 bp, with a GC content of 43.81% and contains 85 predicted open reading frames (ORFs) (Fig. 2A; Table S2). Predicted functional proteins are categorized into six groups: DNA replication, transcription, and translation (6 ORFs); DNA metabolism and modification (6 ORFs); virion structure and assembly (21 ORFs); host-phage interaction proteins (1 ORF); lysis-related proteins (4 ORFs); and proteins with uncharacterized functions (47 ORFs). A single tRNA-encoding region was detected in Tiny’s genome and was identified as tRNA-Arg. To evaluate whether phage Tiny is suitable for practical applications, the presence of harmful trait-related genes and its lifestyle were investigated. The results revealed no detectable genes associated with virulence, antibiotic resistance, or lysogeny. Phage lifestyle prediction performed with the PhageAI platform (33) also confirmed that Tiny is a virulent phage with 99.99% confidence. Together, these findings suggest that phage Tiny could be considered an effective candidate for further evaluation in practical applications.

To further explore its taxonomic classification and comparative relationships with related phages, BLAST search and phylogenetic analyses were conducted. NCBI MegaBLAST analysis revealed that Tiny is an *Escherichia* phage within the family *Chaseviridae* and genus *Carltongylesvirus*, sharing approximately 90% nucleotide sequence similarity with known members of this group. To date, only 17 *Carltongylesvirus* genomes have been deposited in NCBI, highlighting the limited documentation available for this genus. All available genomes were therefore included to investigate the evolutionary relationship of phage Tiny. Members of this genus have previously been described as possessing hybrid features from two viral families: a structure similar to *Myoviridae*, and the presence of a large single subunit RNA polymerase characteristic of *Autographiviridae* (32, 34). Representative viruses from these families were also incorporated into the phylogenetic analysis. The result demonstrated that phage Tiny clustered most closely with four phages, including MLP1, PO103-1, Ecp_YSF, and BSBP01 (Fig. 2B). In particular, *Escherichia* phage MLP1 was identified as the closest relative to Tiny, originating from the same phylogenetic clade and sharing the highest nucleotide identity of 90.39%. The family *Chaseviridae* formed a distinct phylogenetic cluster, separated from T4-like and T7-like viruses, and was most distantly related to the nucleus-forming jumbophage Goslar (35, 36).

We further examined intergenomic similarity among these four closely related phages using VIRIDIC. The results showed that Tiny shared 83%–86% sequence similarity with its nearest relatives (Fig. 2C). Comparative genomic analysis additionally revealed that these phages contain numerous homologous proteins that are differentially oriented and distributed across their genomes (Fig. 2D). According to species classification criteria, a new species is designated when its nucleotide sequence shares less than 95% identity with other known members (37). Therefore, Tiny is classified as a new species within the genus *Carltongylesvirus*.

### Phage Tiny displays a distinct killing pattern in a strain-specific manner

**Fig 3:**
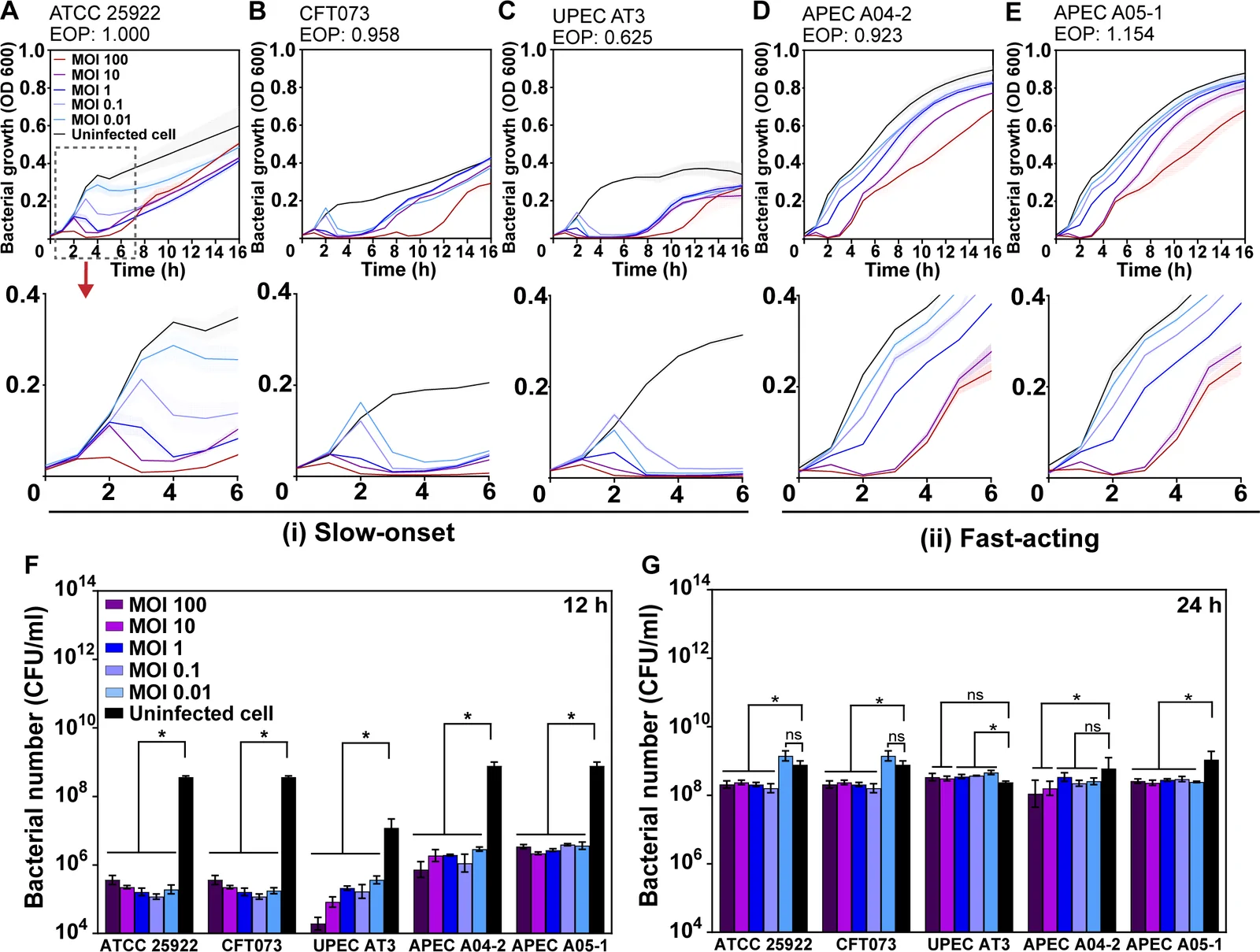
*In vitro* bacterial suppression of phage Tiny against five susceptible *E. coli* strains at different MOIs over a 16 h period. (**A–E**) The killing efficiency of phage Tiny is represented by changes in optical density (OD_600_) of *E. coli* strains, including (**A**) ATCC 25922, (**B**) CFT073, (**C**) UPEC AT3, (**D**) APEC A04-2, and (**E**) APEC A05-1, at different MOIs (0.01–100). (**F and G**) Viable bacterial populations after being co-cultured with phage Tiny for (**F**) 12 h and (**G**) 24 h. Data are presented as the mean ± standard deviation from at least three independent biological replicates. Statistical comparisons between uninfected and phage-treated conditions were analyzed by one-way ANOVA with Dunnett’s test. Statistical significance was defined as “*” at *P*-value ≤0.05, and non-significant differences are indicated as “ns.”

Since the interaction of phage Tiny and its host is optimal at 30°C, we therefore opted to investigate phage dose- and time-dependent killing at 30°C. *In vitro* challenge assays were performed to evaluate phage Tiny against five susceptible *E. coli* strains (Table 1) at multiplicities of infection (MOIs) ranging from 0.01 to 100 over a 16 h period. The killing profiles of phage Tiny were categorized into two distinct patterns: (i) a slow-onset killing pattern (Fig. 3A through C), in which Tiny gradually suppressed bacterial growth and achieved substantial killing after approximately 2 h of incubation; and (ii) a fast-acting killing pattern (Fig. 3D and E), in which Tiny suppressed bacterial growth shortly after infection. Based on these killing patterns, three strains, ATCC 25922 (Fig. 3A), CFT073 (Fig. 3B), and UPEC AT3 (Fig. 3C), were grouped into pattern (i), whereas two APEC strains, A04-2 (Fig. 3D) and A05-1 (Fig. 3E), were categorized into pattern (ii). These strain-dependent killing dynamics may reflect variations in how phage Tiny recognizes and infects its bacterial hosts, which in turn influence the timing and efficiency of its replication.

Despite its ability to inhibit the growth of several *E. coli* strains, resistant populations eventually emerged during treatment and resumed growth during the prolonged treatment (Fig. 3A through E). Notably, resistant populations appeared in the fast-acting killing pattern as early as 3 h after incubation, which was faster than those observed in the slow-onset killing pattern (after 5 h in ATCC 25922 and after 6 h in CFT073 and UPEC AT3). Viable cell counts supported these observations and additionally showed that Tiny still exhibited significant bacterial inhibition at 12 h but was considerably less efficient at 24 h (Fig. 3F and G). In particular, at 24 h and at MOI 0.01, inhibition was too weak to suppress bacterial growth, resulting in bacterial regrowth to a level comparable to the uninfected control (Fig. 3G). However, higher MOIs remained effective and significantly suppressed ATCC 25922, CFT073, APEC A04-2, and A05-1. Taken together, our results indicate that Tiny exhibits markedly stronger bactericidal activity during the early stages of bacterial growth than in the late phase, when resistant bacterial populations emerge and resume growth, particularly at lower doses.

### Phage Tiny exhibits host-dependent adsorption dynamics

Given the strain-dependent killing patterns of Tiny, we hypothesized that these differences arise from variations in phage-host compatibility across distinct phage-host pairs, potentially driven by the diversity of host receptors that determine the initial step of infection. To investigate variation in phage attachment across different bacterial hosts, we conducted adsorption kinetic assays and evaluated the adsorption rate constant (Ka). Based on the killing profiles of Tiny (Fig. 3), two representative strains were selected: UPEC AT3, representing a slow-onset killing pattern, and APEC A05-1, representing a fast-acting killing pattern. Importantly, phage Tiny retained infectivity in both *E. coli* representatives at 30 and 37°C (Fig. 1E; Table 1), allowing comparative evaluation of adsorption kinetics under different temperature conditions.

**Fig 4:**
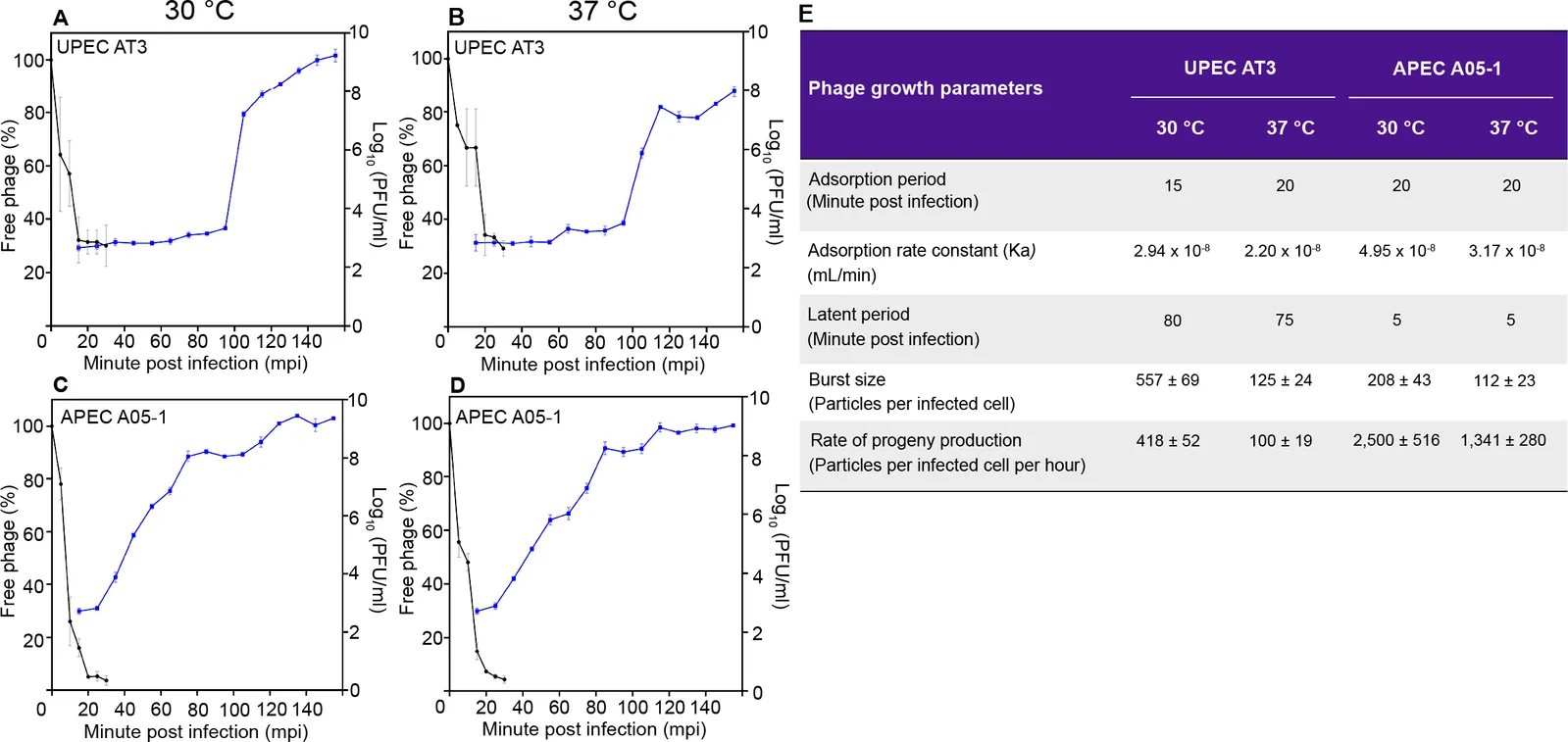
Adsorption kinetics and infection outcomes of phage Tiny in different *E. coli* strains. (**A–D**) Adsorption kinetics, represented as the percentage of free phage (black line; left *y*-axis), and one-step growth curves (blue line; right *y*-axis) of phage Tiny infecting (**A and B**) UPEC AT3 and (**C and D**) APEC A05-1 at 30 or 37°C. Data in panels A–D are presented as the mean ± standard deviation from at least three independent biological replicates. (**E**) Summary of phage growth parameters and infection outcomes determined from the adsorption and lytic kinetics shown in panels A–D.

The adsorption assays revealed that host attachment efficiency is strongly dependent on the *E. coli* strain. Phage Tiny exhibited lower adsorption efficiency toward UPEC AT3, the representative of the slow-onset killing pattern, while demonstrating higher adsorption efficiency toward APEC A05-1, the representative of the fast-acting killing pattern (Fig. 3 and 4). Approximately 68% of Tiny particles adsorbed to UPEC AT3 within 15 mpi at 30°C, after which adsorption reached a plateau (Fig. 4A and E). Similarly, at 37°C, around 65% was observed within 20 mpi before reaching equilibrium (Fig. 4B and E). Only slight differences in adsorption trends were observed between temperatures for the Tiny–UPEC AT3 pair (Fig. 4A and B). Similar adsorption kinetics were also observed in ATCC 25922 (the parental host; slow-onset killing pattern), where around 60% of phages adsorbed within 25 mpi before reaching equilibrium (Fig. S1A). In contrast, Tiny exhibited substantially higher adsorption efficiency toward APEC A05-1, reaching about 95% adsorption at both temperatures, followed by equilibrium after 20 mpi (Fig. 4C through E).

To quantitatively compare adsorption dynamics across strains under different temperature conditions, the adsorption rate constant (Ka) was used for comparison. The constant was calculated by linear regression of ln(Pt/P0) over adsorption time, which represents the rate at which phage particles attach to bacterial cells per unit time (38). We found that the proportion of adsorbed phages correlates with Ka values. Tiny showed lower adsorption rate constants in the low-adsorbing strain UPEC AT3 (2.94 × 10^−8^ mL/min; *R*² = 0.9282 at 30°C and 2.20 × 10^−8^ mL/min; *R*² = 0.8189 at 37°C), whereas higher values were observed in the high-adsorbing strain APEC A05-1 (4.95 × 10^−8^ mL/min; *R*² = 0.9638 at 30°C and 3.17 × 10^−8^ mL/min; *R*² = 0.9457 at 37°C) (Fig. 4E). The lowest adsorption rate constant was observed in the Tiny-ATCC 25922 pair (1.72 × 10^−8^ mL/min; *R*² = 0.9633), which showed the lowest number of adsorbed phages (Fig. S1B). Altogether, these results demonstrate that the efficiency and rate of Tiny adsorption vary across *E. coli* pathotypes, suggesting that differences in host receptor composition contribute to variability in the initial stage of the phage replication cycle.

### Temperature influences infection outcomes across host backgrounds without altering the latent period

Phage-host interactions are shaped by many factors, including receptor recognition, host physiological state, and overall phage-host compatibility, all of which can collectively determine distinct infection outcomes (39–42). To explore the infection outcome of Tiny across different bacterial host backgrounds, we performed one-step growth assays to determine the latent period and burst size for each phage-host pair. The results showed that the one-step growth curve of phage Tiny differed depending on the bacterial strain infected. Based on variation in the latent period (Fig. 4; Fig. S1), the lytic behavior of Tiny could be summarized as follows: (i) Tiny required approximately 70–80 min to complete its replication cycle before releasing virions in a sharp burst (Fig. 4A and B; Fig. S1); and (ii) Tiny exhibited a short latent period of about 5 min, followed by a gradual rise phase lasting approximately 50 min before reaching a plateau (Fig. 4C and D). These one-step growth patterns closely corresponded with the adsorption dynamics and bacterial killing patterns observed among the tested strains. When Tiny exhibited a slower adsorption rate and lower adsorption efficiency, the latent period was prolonged (Fig. 4A and B; UPEC AT3 and Fig. S1A; ATCC 25922) and suppression of bacterial growth was accordingly delayed (Fig. 3A; ATCC 25922 and Fig. 3C; UPEC AT3; a slow-onset killing pattern). In contrast, when Tiny adsorbed rapidly with higher efficiency, it completed the latent phase quickly and progressively released virions during the rise period (Fig. 4C and D; APEC A05-1). This behavior was associated with immediate suppression of bacterial growth (Fig. 3E; APEC A05-1; a fast-acting killing pattern).

Since the interaction between phage Tiny and its host was strongly influenced by temperature, we further examined whether temperature also affects these infection outcomes in each phage-host pair. The results revealed that temperature influenced phage progeny production across the tested pairs, but not the latent duration (Fig. 4E). Tiny produced a larger burst size and a higher rate of progeny production at 30°C than at 37°C in both UPEC AT3 and APEC A05-1 (Fig. 4E). This observation was consistent with the larger plaque size observed at 30°C, indicating enhanced progeny production under this condition (Fig. 1E through G; UPEC AT3 and APEC A05-1). Notably, Tiny produced the highest progeny yield in UPEC AT3 at 30°C, reaching 557 ± 69 particles per infected cell (Fig. 4E), which corresponded to the largest plaque size observed in this host (2.33 ± 0.35 mm; Fig. 1F). Conversely, at 37°C, lower progeny production was associated with smaller plaque sizes of less than 1 mm (Fig. 1G). A similar trend was also observed in APEC A05-1. However, unlike progeny production, the overall timing of the phage replication cycle, as represented by the latent period, remained constant within a certain phage-host pair, regardless of the infection temperatures (Fig. 4A through D). Together, these findings demonstrate that temperature influences the interaction between Tiny and its host, as reflected by changes in progeny yield, but does not significantly alter the duration of the lytic cycle. Therefore, the variation in latent period observed among different *E. coli* pathotypes is more likely driven primarily by phage-host compatibility, which in turn shapes the timing and progression of the replication cycle.

### Single-cell infection analysis reveals morphological dynamics of phage Tiny during extended latent period

Phages with compact genomes are believed to rely heavily on host cellular machinery for efficient replication and therefore frequently encode proteins that modulate fundamental bacterial metabolic pathways (43). Several phage-encoded factors that interfere with host processes have been reported to induce pronounced alterations in bacterial cell morphology, including cell elongation, rounded-shaped cells, or chromosome condensation (25, 30, 44–46). Previously, we exploited such phage-induced morphological changes as a phenotypic guide for identifying a phage-derived antimicrobial (30). However, extending this single-cell analytical approach to phages with compact genomes remains technically challenging, largely due to their relatively short latent periods, which limit the temporal window for capturing and characterizing infection-associated morphological transitions.

Phage Tiny exhibited a prolonged latent period of approximately 70–80 min when infecting several strains, including ATCC 25922 and UPEC AT3 (Fig. 4). Notably, during infection of ATCC 25922, Tiny displayed both the prolonged latent period (70 min) and the lowest burst size (58 ± 6 particles per infected cell, and 50 ± 5 particles per infected cell per hour) (Fig. S1B). This combination presents an intriguing “blind box” that motivates us to further dissect how Tiny interacts with its bacterial host from the initiation of infection through the final lysis event. The extended replication cycle of Tiny therefore provides a valuable temporal window for tracking step-by-step intracellular changes during the infection. In contrast, the shorter latent period observed in APEC strains poses a challenge for visualizing phage-induced morphological transitions, as rapid lysis limits the ability to capture intermediate states. Consequently, the Tiny and ATCC 25922 pair was selected for subsequent single-cell infection analyses.

**Fig 5:**
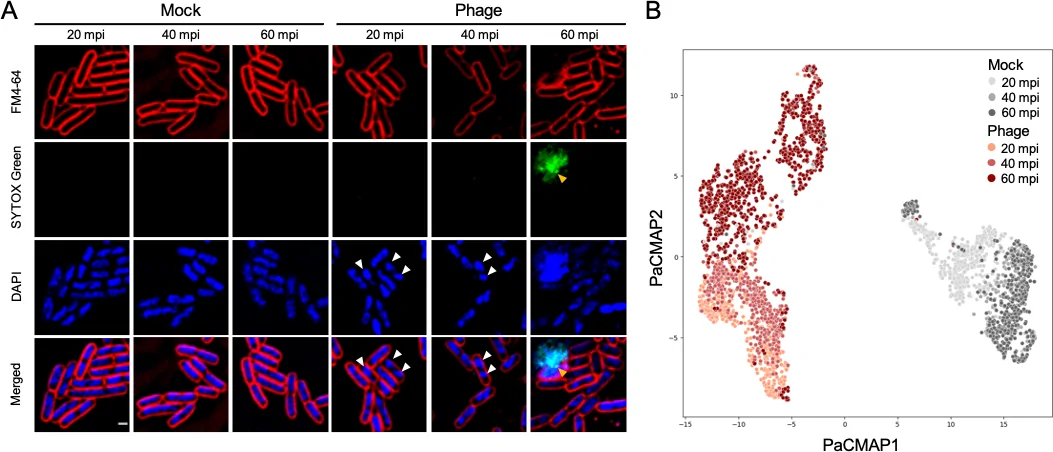
Temporal morphological changes in the representative strain *E. coli* ATCC 25922 during infection by phage Tiny (MOI = 10). (**A**) Time-series images were captured at 20, 40, and 60 mpi to visualize morphological dynamics of phage-infected cells (right panels) and mock-treated controls (left panels) during the extended latent period of the Tiny life cycle. Bacterial cells were stained with FM4-64 (red), DAPI (blue), and permeable cells were positively stained with SYTOX Green (green). During infection, the nucleoids became condensed (white arrows) and host cells were lysed at late infection (yellow arrows). Scale bar equals 1 μm. (**B**) Clusters representing morphological transitions in Tiny-infected cells during the latent period (20, 40, and 60 mpi) are visualized on the Pairwise Controlled Manifold Approximation (PaCMAP). The number of cells of each cluster is designated as follows: (i) mock: 20 mpi (*n* = 558 cells); 40 mpi (*n* = 469 cells); 60 mpi (*n* = 1,095 cells) and (ii) Tiny-infected cells: 20 mpi (*n* = 545 cells); 40 mpi (*n* = 199 cells); 60 mpi (*n* = 584 cells).

We therefore conducted a single-cell infection assay at 20, 40, and 60 min post-infection (mpi) to examine whether (i) Tiny could induce detectable morphological changes comparable to those caused by other phages (25, 30, 47, 48) and (ii) distinct morphological patterns would emerge across different time points relative to uninfected control cells (Mock). The results showed that bacterial morphological alterations were evident at all observed time points (Fig. 5A), indicating that Tiny induces bacterial morphological changes upon infection. Particularly, at 20 mpi, infected cells appeared more compact than control cells, with slightly condensed nucleoids (Fig. 5A; Phage, 20 mpi, white arrows). These changes became more pronounced at 40 mpi, where DNA condensation was clearly visible (Fig. 5A; Phage, 40 mpi, white arrows). By 60 mpi, some cells exhibited lysis (Fig. 5A; Phage, 60 mpi, yellow arrows), while intact cells displayed highly condensed DNA and markedly reduced DAPI intensity relative to earlier infection stages (Fig. 5A; Phage, 60 mpi). These observations likely indicate extensive degradation of the host genome and the near-completion of phage replication (30). Based on these observations, we next performed a single-cell morphological profiling analysis using our previously established single-cell bacterial cytological profiling platform (29) to comprehensively characterize phage-induced morphological changes during infection. Consistent with microscopic observations, the profiling analysis revealed clear separation between phage-infected and mock control cells across all time points (Fig. 5B), confirming that infection-associated morphological signatures were quantitatively detectable. While mock control cells at 20, 40, and 60 mpi clustered closely together, reflecting minimal morphological variation, phage-infected cells formed a continuous trajectory from 20 to 40 to 60 mpi, indicating a progressive temporal shift in cytological profiles during infection. Notably, the cytological profile of infected cells at 40 mpi was slightly displaced from those at 20 mpi, consistent with the corresponding microscopic observations described earlier. This shift became more pronounced at 60 mpi, where infected cells clustered farther from those at 40 mpi, showing a greater degree of morphological deviation than that observed between 20 and 40 mpi. Although adjacent time points are clustered in close proximity, their progressive displacement suggests that phage infection drives dynamic cytological changes throughout the early replication cycle. Collectively, these findings reveal that phage infection induces distinct, time-dependent morphological transitions, providing valuable insight into the temporal progression of the phage replication process at the single-cell level.

## DISCUSSION

With the alarming rise of antibiotic resistance worldwide, the development of effective alternatives to conventional antibiotics has become an urgent priority. Bacteriophages and their genomes represent an underexplored reservoir of potentially novel antimicrobial agents. Here, we report the identification of coliphage Tiny, a new member of the genus *Carltongylesvirus* (Fig. 1 and 2), and show that its infectivity varies depending on both infection temperatures and bacterial host strains, with infection in certain hosts resulting in a prolonged latent duration (Fig. 3 and 4; Table 1). We further reveal, at the single-cell level, that phage Tiny induces temporal bacterial morphological dynamics throughout this extended latent period (Fig. 5), suggesting sequential interference with host physiology as part of its replication cycle prior to cell lysis and providing a broadly applicable framework for dissecting lytic phage biology with high temporal resolution.

Phage adsorption represents a critical initial step that determines the compatibility of phage-host interactions. The process is mediated by the recognition of specific receptors on the host cell surface by phage tail structures, and the efficiency of phage attachment typically depends on the strength and specificity of these receptor-tail interactions (49). In this study, we evaluated the adsorption dynamics of Tiny across different *E. coli* pathotypes at different temperatures. Based on our observations, Tiny exhibited faster attachment to APEC A05-1, reaching an equilibrium stage within 20 min and adsorbing ~90% of phage particles at both 30 and 37°C (Fig. 4C through E). This pattern clearly differed from that observed in UPEC AT3 (Fig. 4A, B, and E) and ATCC 25922 (Fig. S1), both of which exhibited lower adsorption efficiency. Our data therefore demonstrate that the adsorption of Tiny is strain-dependent, likely reflecting variation in receptor recognition caused by differences in cell surface composition among *E. coli* pathotypes. In general, tailed coliphages utilize two classes of receptors: a primary receptor, the enterobacterial common antigen (e.g., O-antigen), which mediates initial attachment, and a secondary receptor, the core lipopolysaccharide (LPS), which facilitates DNA injection (49). Most recently, the BASEL (BActeriophage SElection for your Laboratory) collection has further emphasized the importance of primary and secondary receptor recognition on *E. coli* surfaces among diverse bacteriophage genera (50, 51), providing a valuable framework for host range prediction. However, based on our genome analysis, Tiny could not be assigned to any currently reported members represented in the BASEL collection, suggesting that Tiny may represent a lineage with receptor recognition features that are not yet captured within the current BASEL framework, further underscoring the diversity of phage-host interactions. Previous studies have shown that many phages in the family *Chaseviridae* (Fig. 2) utilize LPS on the bacterial outer membrane as a receptor (52–55). Notably, among the closest relatives of Tiny within the genus *Carltongylesvirus* (Fig. 2), phage MLP1 has been shown to recognize the sugar nucleotide GDP-mannose (GDP-Man) in the O-antigen region within LPS in UPEC 1007 as its primary receptor (54). In contrast, MLP1 loses infectivity toward other UPEC strains with distinct LPS compositions, highlighting the critical role of O-antigen serotypes and LPS structural diversity in host recognition among phages in this genus (54). Given this close relationship, we speculate that Tiny may also utilize a similar cell surface glycan as a primary receptor for host recognition and attachment. This hypothesis is supported by our observation that Tiny can infect *E. coli* strains CFT073 and ATCC 25922, which share the same serotype (O6), but not UTI89, which carries a different serotype (O18) (Table 1). Moreover, our data also suggest that receptor compositions or receptor accessibility would be varied among permissive strains as adsorption efficiency differed substantially in APEC A05-1, UPEC AT3, ATCC 25922 (Fig. 4; Fig. S1). Further investigation will therefore be required to experimentally define its receptors and factors involved in its adsorption efficiency.

Phage infection dynamics are influenced by several factors, including host physiological state and phage-host compatibility, which together lead to diverse infection outcomes reflected by bacterial suppression patterns, burst size, and latent period duration (40, 42). Our data revealed that distinct *E. coli* strains respond differently to Tiny under identical temperature conditions (Fig. 4E; Fig. S1; burst size among tested bacteria). This concept is partially supported by their divergent growth patterns, despite being cultured under similar conditions (Fig. 3A through E), which suggests the distinct physiology of bacterial hosts. Similarly, the same *E. coli* strain can respond differently to phage infection at different temperatures, likely due to its physiology being altered by temperatures. This idea is consistent with our observation that Tiny produces more progeny at 30°C than at 37°C in the same bacterial hosts (Fig. 4E; Fig. S1 burst size at 30°C and 37°C). While the reduced replication efficiency at 37°C may limit the direct application of Tiny for clinical use in patients, its enhanced replication at 30°C suggests that it could be better suited for ecological applications or in environmental reservoirs that are associated with lower temperatures. It is worth noting that, however, the latent period remained unchanged or exhibited minor differences in certain host backgrounds regardless of infection temperatures (Fig. 4E; Fig. S1; latent periods among tested bacteria at 30°C and 37°C). These findings suggest that, differing from other phage-host interactions that rely on the infection temperatures, the latent period of Tiny, similar to its host adsorption, is likely dependent on the certain type of bacterial host, which serves as a crucial factor contributing to the phage-host compatibility.

The latent period can vary across phage-host pairs, reflecting differences in their interaction dynamics (39, 40). It represents an eventual outcome of specific phage-host interplay that is shaped by multiple sequential events, ranging from adsorption and DNA injection to subcellular organization and cell egress (41). Adsorption, as a critical step for entry that warrants the phage-host compatibility, can influence the subsequent replication process within the bacterial host, with phages exhibiting higher adsorption rates, presumably more compatible phage-host pairing, often displaying faster lysis times (56). Consistent with our observations, Tiny exhibited faster and more efficient attachment to the cell surface of APEC A05-1, which is likely to promote earlier initiation of the replication cycle compared to UPEC AT3 (Fig. 4E) and ATCC 25922 (Fig. S1). This, in turn, resulted in more rapid cell lysis, as observed by a shorter latent period, along with increased phage production, as indicated by a greater burst size (Fig. 4E; Fig. S1). Specifically, Tiny displayed a short latent period with high progeny production in the fast-adsorbing strain APEC A05-1, whereas it exhibited an extended latent period with lower progeny production in the slow-adsorbing strains UPEC AT3 and ATCC 25922. This reduced adsorption efficiency may reflect partial incompatibility of phage-host interactions, thereby contributing to both the extended latent period and reduced progeny yield, potentially due to a prolonged duration required for effective host takeover. This observation is consistent with our previous study (48), in which two distinct jumbophages infecting the same bacterial host exhibited different life cycle durations, where the phage with a higher adsorption rate completed its life cycle more rapidly and produced greater progeny. However, the latent period is not solely determined by the adsorption kinetics. We have also demonstrated that the increased independence in phage maturation is potentially associated with a shorter life cycle (48). Altogether, even though the adsorption rate is a key determinant of phage-host compatibility, post-entry processes after DNA injection into the host cell are also influenced by additional factors, including phage-host mechanistic interactions and anti-phage defense systems, which, in turn, collectively shape the duration of the latent period and the efficiency of virion production. Nevertheless, the relative contribution of these factors remains elusive and requires further investigation.

Compared with other members within the family *Chaseviridae*, previous reports describe that latent periods can be varied from 20 to 35 min among members, accompanied by considerable variation in their burst sizes (57–60) (Table S3). Several members also display prolonged latent stages similar to Tiny in specific *E. coli* strains. For example, *Escherichia* phage vB_EcoM-4HA13 exhibits a latent period of up to 90 min with ~55 progeny production (61). Similarly, *Escherichia* phage ST32 exhibited a prolonged latent stage (~102 min) at 20°C with a relatively high burst size (602 ± 159 virions per infected cell), whereas infections at higher temperatures (30°C–37°C) yield shorter latent periods (~50–60 min) but reduced progeny yields (~2–64 virions per infected cell) (62). Together, these findings highlight the substantial variability in latent periods and burst sizes within this viral family that is likely dependent on specific phage-host interplay. Importantly, no studies have yet elucidated why certain members require such extended replication periods for successful replication. One of the possible explanations related to this unusual prolonged latent duration would be the presence of anti-phage defense mechanisms. However, our study did not address the impact of differences in anti-phage defense systems among *E. coli* pathotypes on phage growth parameters, and there is currently limited information referring to defense systems targeting phages within the family *Chaseviridae*. Therefore, whether anti-phage defense systems exist within host genomes and modulate with the phage replication cycle, resulting in the variation in infection outcomes in different *E. coli* pathotypes, requires further investigations.

To guarantee successful replication, phages hijack host cellular resources and manipulate essential metabolic and regulatory pathways (63). These processes in turn induce distinct morphological alterations in infected cells throughout the infection stages (25, 47). Previously, we established a phage-induced morphological analysis to dissect the morphological changes induced by phages and to elucidate the phage hijacking mechanisms that sequentially occur prior to lysis, a process we refer to as the “Mechanism of pre-killing (MOK)” (25, 30, 47, 48). Since this platform operates on the principles of BCP, which characterizes the signature morphological phenotypes elicited by antibiotic treatment, it enables the identification of the host cellular pathway affected by the phage and also led to the discovery of phage-derived proteins that exhibit antibiotic-like activity (30). However, previous studies have offered only static snapshots of the infection process, leaving the temporal dynamics of these morphological changes largely unexplored. In this study, the relatively long latent period exhibited by Tiny when infecting particular *E. coli* strains distinguishes it from many compact-genome phages and provides an advantageous system for investigating temporal morphological changes during infection. Using the Tiny-ATCC 25922 model, our analyses reveal that Tiny induces a progressive and continuous morphological shift in bacterial cells from the onset of infection through cell lysis (Fig. 5). Unlike phage-induced morphological changes previously observed in other phages that simply match established BCP signatures (25, 30), Tiny exhibited a distinct cytological response. For example, the previous study showed that the jumbo vibriophage KVP40 transforms *V. parahaemolyticus* cells from rod-shaped toward rounded forms during a 120 min infection cycle. This phenotype closely resembles that caused by the cell wall-targeting antibiotic mecillinam and was traced to the activity of a phage-encoded lytic transglycosylase that compromises the bacterial cell membrane (30). In contrast, the overall morphological transitions induced by Tiny did not resemble any single signature previously reported in our BCP database. This is not surprising and likely attributed to the fact that phages usually exert multiple MOKs simultaneously, resulting in mixed apparent phenotypes when no single morphological signature predominates. This concept is widely supported by several transcriptomic analyses reporting extensive temporal reprogramming of host genes and the phage gene expression involved in phage-host interactions prior to lysis (64–66). Further extension of our BCP database to include all possible bacterial morphological responses triggered by combinations of antibiotics will be required to facilitate the identification of cellular targets perturbed by phages. In parallel, because phage genomes often contain numerous unknown genes due to limited reference genomic data, the development of improved gene annotation tools, coupled with expanded omics data sets (e.g., RNA-seq, proteomics), will be crucial for uncovering novel antimicrobials embedded within these underexplored genetic resources. Altogether, our findings provide supporting evidence that phage infection not only reprograms host gene expression but also drives temporal phenotypic alterations of bacteria throughout the phage infection cycle.

## MATERIALS AND METHODS

### Bacterial growth conditions and phage preparation

Bacterial strains used in this study are listed in Table 1. All strains were routinely cultured in Luria-Bertani (LB) broth or on LB agar, composed of 10 g/L tryptone (Himedia, India), 10 g/L NaCl (KemAus, Australia), 5 g/L yeast extract (Himedia, India), and 1.5% (wt/vol) agar (Difco, USA), and incubated at 37°C.

*Escherichia* phage Tiny was preserved in 25% glycerol as a freezer stock in our laboratory collection. To prepare a fresh high-titer stock, phage Tiny was propagated using *E. coli* ATCC 25922 as the host strain. The phage was recovered from the glycerol stock by streaking onto LB agar overlaid with a 0.7% soft-agar lawn of the host strain and incubating the plate at 30°C for 16–18 h. After incubation, a single plaque was picked and soaked in 200 μL of SM buffer overnight at 4°C. Subsequently, 50 μL of the recovered phage and 200 μL of log-phase host culture were added onto separate areas of an LB agar plate, followed by overlaying with 5 mL of 0.7% soft agar and gently swirling to ensure even coverage. The plates were incubated at 30°C overnight. Plates showing clear to confluent lysis were flooded with 5 mL SM buffer for at least 5 h, and the phage lysate was collected. The lysate was centrifuged at 8,000 rpm for 5 min and filtered through a 0.45 µm nylon syringe filter. Ten-fold serial dilutions of the lysate were prepared and spotted onto 0.7% DLA to determine the phage titer (PFU/mL). High-titer lysates were stored at 4°C until use in subsequent experiments.

### Morphological and biological characterizations

A full plate assay was performed to observe plaque morphology on bacterial lawns. Briefly, 200 μL of log-phase bacterial culture and 50 μL of 10-fold diluted phage lysate were added directly onto the LB agar plate. Pre-warmed 0.7% soft agar was then poured over the mixture and the plate gently swirled. Plates were incubated at 30°C or 37°C for 18 h. Plates displaying clearly separated plaques were imaged using the ImageQuant Analyzer, and plaque sizes were measured using Fiji software (67).

The structure of phage Tiny was visualized using TEM. The sample preparation was carried out following the method reported by Naknaen et al. (48). Briefly, 5 mL of pre-warmed 0.7% soft agar containing 200 μL of *E. coli* ATCC 25922 log-phase culture was poured onto LB agar. After the agar solidified, 20 μL of high-titer phage lysate was spotted onto the surface and incubated at 30°C overnight. The large clear spot was picked and soaked in 500 μL SM buffer overnight at 4°C. The phage lysate was centrifuged at 10,000 rpm for 10 min to remove excess soft agar and then filtered through a 0.45 µm syringe filter. The purified phage lysate was dropped onto the center of a carbon-coated TEM grid and stained with 2% uranyl acetate. The morphological structure of phage Tiny was then observed under TEM (HITACHI model HT7700).

The effects of pH and temperature on phage viability were evaluated following the protocol previously described by Khunti et al. (68). In brief, pH tolerance was evaluated by incubating phage Tiny (10^7^ PFU/mL) in SM buffer adjusted to various pH levels (2, 4, 6, 7, 8, 10, and 12) at 30°C. Temperature tolerance was tested by incubating phage Tiny (10^7^ PFU/mL) in SM buffer at 4°C, 20°C, 25°C, 30°C, 37°C, 50°C, 60°C, and 70°C. After 1 h of incubation, the final phage concentrations (PFU/mL) were determined, and the percentage of phage survival was calculated.

### Phage DNA isolation, whole-genome sequencing, and bioinformatic analysis

Phage DNA was isolated from a high-titer lysate using the phenol-chloroform-isoamyl alcohol method (68). The purified DNA was sequenced on an Illumina MiSeq platform, and raw reads were assembled using SPAdes (v.3.15.3). Genome annotation of the assembled contig was performed with Pharokka (v.1.3.2). Moreover, functional proteins were predicted using the NCBI database and NCBI conserved domains, and the presence of tRNA was identified using tRNAscan-SE. The complete genome map of phage Tiny was visualized using Proksee web server. To screen whether Tiny harbors genes associated with bacterial virulence or antibiotic resistance, its genome was analyzed using VirulenceFinder, ResFinder, and AMRFinderPlus (NCBI Antimicrobial Resistance Gene Finder). Phage lifestyle was also predicted using the PhageAI platform (33). To examine the evolutionary relationships and ancestry of phage Tiny, a phylogenetic tree was constructed using VICTOR and visualized with the iTOL web server. Four phylogenetically related bacteriophages within the same genus as Tiny were selected to further evaluate their genomic similarity and distance. Intergenomic similarity analyses between phage Tiny and four related phages, including MLP1 (NC_079176.1), PO103-1 (NC_079175.1), Ecp_YSF (OR327751.1), and BSBP01 (PQ231202.1), were performed using VIRIDIC. In addition, multiple genome comparisons among these phages were generated and visualized using DiGAlign.

### Host range determination and EOP assay

Host range determination of phage Tiny was assessed using 27 *E. coli* strains (Table 1). Briefly, 5 μL of high-titer phage lysate was spotted onto bacterial lawns, and the plates were incubated at 30°C or 37°C overnight. For the EOP assay, *E. coli* strains that exhibited clear lysis were subsequently selected. Ten-fold serial dilutions of phage Tiny were spotted onto bacterial lawns and incubated at 30°C or 37°C. The number of observed plaques was counted and expressed as PFU/mL, and the EOP values were calculated as previously described (68).

### Killing activity of phage Tiny

Five *E. coli* strains, including ATCC 25922, CFT073, UPEC AT3, APEC A04-2, and APEC A05-1, were grown to OD_600_ of 0.4 and diluted 10-fold to obtain a final cell concentration of approximately 10^7^ CFU/mL. Phage Tiny was added to 96-well plates containing the bacterial cultures at different MOIs ranging from 0.01 to 100, followed by incubation at 30°C. Bacterial growth (OD_600_) was detected every 10 min for 16 h using a microplate reader. To further assess the bactericidal activity of phage Tiny, viable cell counts (CFU/mL) were measured. Bacterial cultures were mixed with phage Tiny at MOIs of 0.01–100 and incubated at 30°C. At 12 and 24 h, samples were collected, serially diluted, and plated to quantify surviving cells. Bacterial number (CFU/mL) was compared with those of uninfected control. All experiments were performed in triplicate.

### Adsorption kinetic assay

This assay was carried out using the protocol reported by Kongsomboonchoke et al. (31). Initially, the exponential growth phase of *E. coli* strains (OD_600_ ≈ 0.4) was co-cultured with phage Tiny at MOI 0.01 and incubated at either 30°C or 37°C. During incubation, both phage and bacterial concentrations were enumerated at 0 min. Subsequently, 200 µL of the co-culture was collected at 5 min intervals for 40 min and filtered through a 0.45 µm syringe filter to obtain the phage lysate. Serial dilutions of the phage lysate were then prepared and spotted onto 0.7% DLA to quantify the number of remaining free phage over time. The adsorption kinetics of Tiny-*E. coli* interactions were plotted as the proportion of free phages (%) remaining over time to determine the adsorption time (t), defined as the point at which phage adsorption reached equilibrium. Moreover, the adsorption rate constant (Ka) was calculated by fitting a linear regression of ln(Pt/P0) across adsorption time (t), where Pt and P0 represent the free phage titers at time t and at 0 min, respectively, according to the following formula (38):


Adsorption rate constant (Ka)=1/Nt×ln(Pt/P0)

where Ka is the adsorption rate constant (mL/min), N is the concentration of bacterial cells (CFU/mL), t is adsorption time, defined as the point at which phage adsorption reached equilibrium, P0 is the initial free phage titer (PFU/mL) at 0 min, and Pt is the free phage titer at time t (PFU/mL).

### One-step growth assay

The one-step growth assay was performed as previously described (31) to characterize the lytic replication cycle of phage Tiny and to determine the number of progeny virions released per infected cell. As a first step, the absorption assay was carried out to allow the phage to attach to the surface receptors of the bacterial cells within a short incubation period. Exponential-phase cultures (OD_600_ ≈ 0.4) of each *E. coli* strain were mixed with phage Tiny at an MOI of 0.01 in a final volume of 1 mL LB broth. The mixture was briefly vortexed and incubated at 30°C or 37°C for 15 min. Initial phage and bacterial concentrations were also quantified.

After the adsorption step, the culture was centrifuged at 9,000 rpm for 1 min, and the supernatant and bacterial pellet were separately collected. To determine the number of unattached phages, the supernatant was filtered through a 0.45 µm syringe filter to obtain the phage lysate. Serial dilutions of the lysate were prepared and spotted onto a lawn of the host bacteria to enumerate the number of unattached phages (PFU/mL). The number of phages that successfully infected bacterial cells was calculated as the difference between the initial phage population and the number of unattached phages remaining in the supernatant.

Meanwhile, the bacterial pellet was washed with LB broth to remove remaining unattached phages and then centrifuged. The pellet was resuspended in 10 mL of LB broth and mixed thoroughly. The culture was incubated at 30°C or 37°C for at least 180 min. At 10 min intervals, 200 µL aliquots of the culture were collected and immediately filtered through a 0.45 µm syringe filter. The phage concentration at each time point was determined using the spot test method. The one-step growth curve was generated by plotting phage concentrations (PFU/mL) over time to characterize the lytic replication cycle, including the latent, rise, and plateau stages.

The burst size, defined as the number of progeny Tiny phage particles produced per infected cell, and the rate of progeny production, defined as the number of progeny released per bacterium per hour, were calculated using the following formula:

Burst size (particles per infected cell) = (Average phage count at plateau stages − Average phage count at latent stages)/Phage number that successfully infected bacterial cells

Rate of progeny production (particles per infected cell per hour) = Burst size/latent period (hour)

### Single-cell infection assay

The log-phase culture of *E. coli* ATCC 25922 was prepared by subculturing the overnight culture at a 1:100 dilution. The day culture was then grown until OD_600_ reached 0.4 by shaking at 250 rpm, 30°C. Then, the bacterial cells were infected with the high-titer phage lysate at MOI 10. At desired time points: 20, 40, and 60 mpi, 500 μL of infected cells were incubated with fluorescence dyes DAPI (2 μg/mL), FM4-64 (1 μg/mL), and SYTOX Green (0.5 μM) for 1 min. The infected cells were then harvested by centrifugation at 10,000 rpm for 1 min. Then, the stained infected cells were loaded onto an agarose pad (1.2% agarose with 20% LB) on concave glass slides. The bacterial cell morphological responses were then observed by visualizing the samples under a Delta Vision ultra-high-resolution microscope. Experimental settings and imaging parameters were constant throughout every experiment.

### Single-cell bacterial cytological profile analysis

Prior to downstream image analysis, raw images from the fluorescence microscope are pre-processed into an appropriate image format in ImageJ software (67). Then, cell membranes and nuclei are segmented and subsequently analyzed using CellProfiler 4.0 software for cell feature extraction. Previously reported 62 morphological features are selected for bacterial cytological profile analysis (29). Statistical analysis is carried out using the scikit-learn library in Python as previously described. Briefly, cell profile data are transformed and normalized with cube root transformation and Z-score normalization. Outliers, such as incomplete cells along the image edge and abnormal image segmentation, are removed with a 10% cutoff using hierarchical density-based spatial clustering of applications with noise (29). Finally, the dimension of the data set is reduced and then visualized with an unsupervised data dimension reduction method, Pairwise Controlled Manifold Approximation (69).

## Data Availability

The complete genome sequence of phage Tiny is publicly available in the NCBI database under accession number PX572931.

## SUPPLEMENTAL MATERIAL

Supplemental Material spectrum.04159-25-s0001.docx Figure S1 and Tables S1 to S3.

ASM does not own the copyrights to Supplemental Material that may be linked to, or accessed through, an article. The authors have granted ASM a non-exclusive, world-wide license to publish the Supplemental Material files. Please contact the corresponding author directly for reuse.
