# Supplementary material for: Temperature-dependent coliphage induces distinct temporal bacterial morphological dynamics during infection
Source: Microbiol Spectr. 2026 May 27;14(7):e04159-25. doi: 10.1128/spectrum.04159-25 (PMC13340011; doi:10.1128/spectrum.04159-25)
Supplement: Supplemental Material — Figure S1 and Tables S1 to S3. [file spectrum.04159-25-s0001.docx]

**Supplemental Material**

**Temperature-dependent coliphage induces distinct temporal bacterial morphological dynamics during infection**

**Jiranan Pattano,^1^ Filosofia F. T. A. Prasasti,^2^ Songphon Buddhasiri,^3^ Patiphan Khunti,^1^ Panupon Mongkolkarvin^4^, Parameth Thiennimitr,^4,5^ Poochit Nonejuie,^2^ and Vorrapon Chaikeeratisak^1^**

^1^Department of Biochemistry, Faculty of Science, Chulalongkorn University, Bangkok, Thailand.

^2^Center for Advanced Therapeutics, Institute of Molecular Biosciences, Mahidol University, Nakhon Pathom, Thailand

^3^Veterinary Public Health and Food Safety Centre for Asia Pacific, Faculty of Veterinary Medicine, Chiang Mai University, Chiang Mai, Thailand

^4^Department of Microbiology, Faculty of Medicine, Chiang Mai University, Chiang Mai,

Thailand

^5^Center of Excellence in Microbial Diversity and Sustainable Utilization, Chiang Mai University, Chiang Mai, Thailand

**Corresponding author.***E-mail address:*

Vorrapon Chaikeeratisak, vorrapon.c@chula.ac.th

**Supplemental Figure**

**
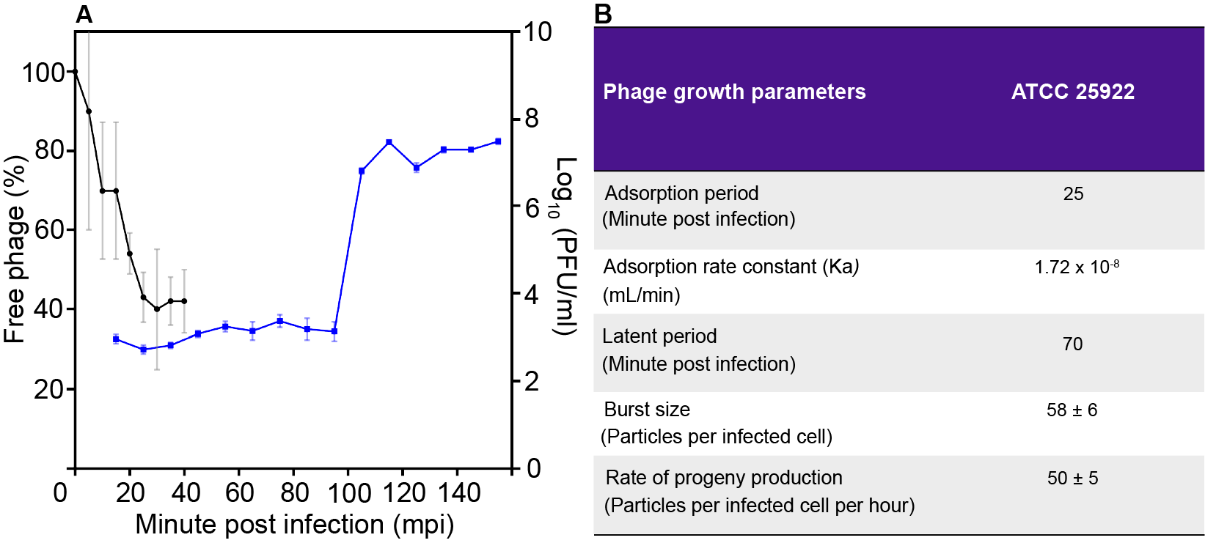
**

**Fig. S1** Adsorption kinetics and lytic life cycle of phage Tiny in *E. coli* strain ATCC 25922. (A) Adsorption kinetic, represented as the percentage of free phage (black line; left y-axis), and one-step growth curve (blue line; right y-axis) of phage Tiny infecting ATCC 25922 at 30°C. Data is presented as the mean ± standard deviation from at least three independent biological replicates. (B) Summary of phage growth parameters and infection outcomes determined from the adsorption and lytic kinetics shown in A.

**Supplemental Table**

**Table S1** Antimicrobial susceptibility testing of 15 avian pathogenic *Escherichia* coli (APEC) isolates

| **APEC strains** ^a^ | **MIC (µg/ml)** ^b^ | | | |
| --- | --- | --- | --- | --- |
|  | **Ceftriaxone** | **Ciprofloxacin** | **Colistin** | **Gentamicin** |
| DO1A | < 0.125 (S) | >2 (R) | 1 (S) | 1 (S) |
| DO7A | 0.25 (S) | 0.5 (I) | 1 (S) | 1 (S) |
| D31D | 0.25 (S) | 0.25 (S) | 2 (S) | >32 (R) |
| D511A | < 0.125 (S) | 0.5 (I) | 2 (S) | >32 (R) |
| D713A | < 0.125 (S) | >2 (R) | 1 (S) | 2 (S) |
| D4416D | < 0.125 (S) | <0.031 (S) | 1 (S) | 2 (S) |
| A04-2 | < 0.125 (S) | 0.125 (S) | 1 (S) | 2 (S) |
| A05-1 | < 0.125 (S) | 0.125 (S) | 1 (S) | 1 (S) |
| A11-1 | < 0.125 (S) | >2 (R) | 1 (S) | 2 (S) |
| A15-1 | < 0.125 (S) | >2 (R) | 1 (S) | 1 (S) |
| B01-1 | < 0.125 (S) | 2 (R) | 1 (S) | >32 (R) |
| B09-4 | >8 (R) | 0.250 (S) | 1 (S) | >32 (R) |
| E02-5 | < 0.125 (S) | 2 (R) | 1 (S) | >32 (R) |
| E05-2 | < 0.125 (S) | 2 (R) | 1 (S) | >32 (R) |
| F01-5 | 0.25 (S) | 0.25 (S) | 1 (S) | 1 (S) |

^a^ Antimicrobial susceptibility testing was performed to distinguish APEC isolates and determine drug resistance profiles, whereas the susceptibility of UPEC isolates was previously reported by Kongsomboonchoke *et al* (31).

^b^ Antimicrobial susceptibility was interpreted as Susceptible (S), Intermediate (I), or Resistant (R) based on the CLSI M100 guidelines.

**Table S2** Predicted functions of ORFs in the genome of phage Tiny (NCBI accession number: PX572931)

| **CDS** | **Position** | | **Predicted functions** | **Product size** | **Sequence similarity** | **Accession no.** | **Identity**  **(%)** | **Coverage**  **(%)** | **E-value** |
| --- | --- | --- | --- | --- | --- | --- | --- | --- | --- |
|  | **Start** | **End** |  |  |  |  |  |  |  |
| 1 | 30 | 152 | Hypothetical protein | 40 | Shigella phage vB_Shi_2KOFFS | XUU32474.1 | 95 | 100 | 2.00E-18 |
| 2 | 303 | 1421 | Ribonucleoside diphosphate reductase small subunit | 372 | Yersinia phage PYps16N | YP_010844744.1 | 99.46 | 100 | 0 |
| 3 | 1424 | 1981 | Endolysin | 186 | Yersinia phage PYps3T | YP_010844576.1 | 98.92 | 100 | 2.00E-135 |
| 4 | 2000 | 2341 | Holin/anti-holin | 133 | Yersinia phage PYps3T | YP_010844575.1 | 100 | 100 | 5.00E-77 |
| 5 | 2341 | 2871 | Tail fiber assembly protein | 180 | Escherichia phage BSBP01 | XFC50487.1 | 90.34 | 98 | 2.00E-113 |
| 6 | 2852 | 4204 | Tail fiber protein | 450 | Escherichia phage Ecp_YSF | WMM91820.1 | 91.11 | 100 | 0 |
| 7 | 4214 | 5605 | Tail fiber protein | 463 | Escherichia phage Ecp_YSF | WMM91819.1 | 92.22 | 100 | 0 |
| 8 | 5605 | 6804 | Tail fiber protein | 399 | Yersinia phage PYps4T | QQO91166.1 | 95.99 | 100 | 0 |
| 9 | 6801 | 7445 | Hypothetical protein | 214 | Escherichia phage MLP1 | YP_010844441.1 | 97.2 | 100 | 8.00E-150 |
| 10 | 7454 | 8608 | Hypothetical protein | 384 | Escherichia phage MLP1 | YP_010844440.1 | 97.65 | 100 | 0 |
| 11 | 8584 | 8955 | Hypothetical protein | 123 | Escherichia phage MLP1 | YP_010844439.1 | 95.93 | 100 | 8.00E-82 |
| 12 | 8955 | 9746 | Baseplate spike | 263 | Escherichia phage PO103-1 | YP_010844378.1 | 98.48 | 100 | 0 |
| 13 | 9715 | 10077 | Virion structural protein | 120 | Escherichia phage PO103-1 | YP_010844379.1 | 95.83 | 100 | 4.00E-81 |
| 14 | 10074 | 10976 | Tail protein | 300 | Escherichia phage BSBP01 | XFC50496.1 | 94 | 100 | 0 |
| 15 | 10976 | 12073 | Baseplate hub | 365 | Escherichia phage BSBP01 | XFC50497.1 | 98.08 | 100 | 0 |
| 16 | 12149 | 15838 | Virion structural protein | 1229 | Escherichia phage MLP1 | YP_010844434.1 | 88.57 | 100 | 0 |
| 17 | 16007 | 16465 | Tail tube protein | 152 | Yersinia phage PYps23T | YP_010844561.1 | 99.34 | 100 | 3.00E-107 |
| 18 | 16481 | 16816 | Virion structural protein | 111 | Yersinia phage PYps23T | YP_010844642.1 | 98.2 | 100 | 2.00E-73 |
| 19 | 16946 | 18394 | Tail sheath | 482 | Yersinia phage PYps23T | YP_010844641.1 | 93.98 | 100 | 0 |
| 20 | 18397 | 18912 | Tail completion | 171 | Yersinia phage PYps16N | YP_010844725.1 | 96.49 | 100 | 6.00E-120 |
| 21 | 18912 | 19328 | Head-tail adaptor protein | 138 | Escherichia phage PO103-1 | YP_010844385.1 | 97.83 | 100 | 5.00E-98 |

**Table S2** Predicted functions of ORFs in the genome of phage Tiny

| **CDS** | **Position** | | **Predicted functions** | **Product size** | **Sequence similarity** | **Accession no.** | **Identity**  **(%)** | **Coverage**  **(%)** | **E-value** |
| --- | --- | --- | --- | --- | --- | --- | --- | --- | --- |
|  | **Start** | **End** |  |  |  |  |  |  |  |
| 22 | 19274 | 19663 | Virion structural protein | 129 | Escherichia phage phiEcoM-GJ1 | YP_001595450.1 | 95.35 | 100 | 6.00E-86 |
| 23 | 19664 | 20104 | Hypothetical protein | 146 | Staphylococcus phage Pel53 | XDJ02288.1 | 94.52 | 100 | 7.00E-86 |
| 24 | 20151 | 21158 | Major head protein | 355 | Escherichia phage phiEcoM-GJ1 | YP_001595448.1 | 97.61 | 100 | 0 |
| 25 | 21325 | 21798 | Virion structural protein | 157 | Escherichia phage vB_EcoP_Bp7 | YP_010844849.1 | 94.87 | 99 | 1.00E-100 |
| 26 | 21808 | 22947 | Head maturation protease | 372 | Escherichia phage BSBP01 | XFC50510.1 | 94.62 | 98 | 0.00E+00 |
| 27 | 22841 | 24160 | Portal protein | 439 | Escherichia phage Mangalitsa | YP_009850526.1 | 99.32 | 100 | 0.00E+00 |
| 28 | 24160 | 24372 | Major head subunit precursor | 70 | Yersinia phage PYps3T | YP_010844550.1 | 91.43 | 100 | 7.00E-39 |
| 29 | 24374 | 26392 | Terminase large subunit | 672 | Escherichia phage vB_EcoP_Bp7 | YP_010844845.1 | 97.47 | 100 | 0 |
| 30 | 26412 | 26597 | Hypothetical protein | 61 | Escherichia phage MLP1 | YP_010844491.1 | 90.16 | 100 | 3.00E-33 |
| 31 | 26652 | 26981 | Hypothetical protein | 109 | Escherichia phage FXie-2024a | WWE96966.1 | 98.17 | 100 | 2.00E-71 |
| 32 | 27026 | 27478 | dUTPase | 150 | Escherichia phage Ecp_YSF | WMM91792.1 | 90 | 100 | 2.00E-95 |
| 33 | 27453 | 28160 | Deoxynucleoside monophosphate kinase | 235 | Escherichia phage BSBP01 | XFC50517.1 | 71.67 | 97 | 6.00E-116 |
| 34 | 28078 | 28833 | ATP-dependent DNA ligase | 251 | Escherichia phage FXie-2024a | WWE96970.1 | 98.01 | 100 | 0 |
| 35 | 28823 | 29347 | HNH endonuclease | 174 | Escherichia phage SKA49 | YP_010844780.1 | 98.85 | 100 | 6.00E-123 |
| 36 | 29359 | 30420 | Exonuclease | 353 | Escherichia phage BSBP01 | XFC50520.1 | 93.2 | 100 | 0 |
| 37 | 30428 | 31294 | Hypothetical protein | 288 | Yersinia phage PYps3T | YP_010844540.1 | 95.83 | 100 | 0 |
| 38 | 31323 | 31595 | Hypothetical protein | 90 | Escherichia phage FXie-2024a | WWE96974.1 | 95.56 | 100 | 2.00E-53 |
| 39 | 31595 | 33508 | DNA polymerase | 637 | Yersinia phage PYps16N | YP_010844703.1 | 94.35 | 100 | 0 |
| 40 | 33596 | 35362 | DNA primase/helicase | 588 | Escherichia phage vB_EcoM_JL1 | WPF71428.1 | 98.13 | 100 | 0 |
| 41 | 35363 | 36013 | Thymidylate synthase | 216 | Escherichia phage vB_EcoM_JL1 | WPF71429.1 | 98.15 | 100 | 3.00E-159 |
| 42 | 36070 | 36291 | Hypothetical protein | 73 | Escherichia phage flopper | YP_009855617.1 | 94.74 | 100 | 4.00E-41 |

**Table S2** Predicted functions of ORFs in the genome of phage Tiny

| **CDS** | **Position** | | **Predicted functions** | **Product size** | **Sequence similarity** | **Accession no.** | **Identity**  **(%)** | **Coverage**  **(%)** | **E-value** |
| --- | --- | --- | --- | --- | --- | --- | --- | --- | --- |
|  | **Start** | **End** |  |  |  |  |  |  |  |
| 43 | 36278 | 36445 | Hypothetical protein | 55 | Yersinia phage PYps23T | YP_010844613.1 | 96.36 | 100 | 2.00E-31 |
| 44 | 36580 | 36717 | Hypothetical protein | 45 | No significant similarity found |  |  |  |  |
| 45 | 36772 | 36867 | Hypothetical protein | 31 | Escherichia phage Ecp_YSF | WMM91880.1 | 80.65 | 100 | 7.00E-09 |
| 46 | 36845 | 37042 | Hypothetical protein | 65 | Shigella phage vB_Shi_2KOFFS | XUU32424.1 | 66.67 | 42 | 0.005 |
| 47 | 37498 | 37734 | Rz-like spanin | 78 | Yersinia phage PYps3T | YP_010844530.1 | 94.87 | 100 | 2.00E-34 |
| 48 | 37743 | 37883 | Hypothetical protein | 46 | Escherichia phage Mangalitsa | YP_009850506.1 | 100 | 100 | 6.00E-23 |
| 49 | 38688 | 38792 | Hypothetical protein | 34 | No significant similarity found |  |  |  |  |
| 50 | 38859 | 38969 | Hypothetical protein | 36 | No significant similarity found |  |  |  |  |
| 51 | 39065 | 39154 | Hypothetical protein | 29 | No significant similarity found |  |  |  |  |
| 52 | 39179 | 39517 | Hypothetical protein | 112 | Escherichia phage Mangalitsa | YP_009850502.1 | 100 | 100 | 4.00E-74 |
| 53 | 39642 | 40316 | Hypothetical protein | 224 | Escherichia phage ST32 | YP_009790691.1 | 100 | 100 | 5.00E-155 |
| 54 | 40318 | 40512 | Hypothetical protein | 64 | Yersinia phage PYps23T | YP_010844607.1 | 100 | 100 | 2.00E-37 |
| 55 | 40605 | 40925 | Hypothetical protein | 106 | Escherichia phage MLP1 | YP_010844471.1 | 95.28 | 100 | 9.00E-68 |
| 56 | 41791 | 42027 | Hypothetical protein | 78 | Escherichia phage BSBP01 | XFC50450.1 | 98.72 | 100 | 1.00E-51 |
| 57 | 42020 | 42568 | Anti-restriction protein | 182 | Escherichia phage Mangalitsa | HGD9033445.1 | 96.7 | 100 | 2.00E-120 |
| 58 | 42555 | 42833 | Hypothetical protein | 92 | Escherichia phage Ecp_YSF | YP_009850494.1 | 96.7 | 100 | 1.00E-53 |
| 59 | 42854 | 43249 | Endolysin | 131 | Escherichia phage Mangalitsa | YP_009850492.1 | 96.92 | 99 | 4.00E-90 |
| 60 | 43249 | 43524 | Hypothetical protein | 91 | Escherichia phage BSBP01 | XFC50454.1 | 97.80 | 100 | 2.00E-55 |
| 61 | 43521 | 43754 | Hypothetical protein | 77 | Escherichia phage phiEcoM-GJ1 | YP_001595414.1 | 97.4 | 100 | 9.00E-49 |
| 62 | 43751 | 44110 | Hypothetical protein | 119 | Yersinia phage PYps23T | YP_010844597.1 | 94.12 | 100 | 9.00E-77 |
| 63 | 44163 | 44639 | Hypothetical protein | 158 | Escherichia phage SKA49 | YP_010844759.1 | 94.3 | 100 | 2.00E-103 |

**Table S2** Predicted functions of ORFs in the genome of phage Tiny

| **CDS** | **Position** | | **Predicted functions** | **Product size** | **Sequence similarity** | **Accession no.** | **Identity**  **(%)** | **Coverage**  **(%)** | **E-value** |
| --- | --- | --- | --- | --- | --- | --- | --- | --- | --- |
|  | **Start** | **End** |  |  |  |  |  |  |  |
| 64 | 44629 | 44748 | Hypothetical protein | 39 | Yersinia phage PYps23T | YP_010844595.1 | 94.87 | 100 | 3.00E-19 |
| 65 | 44745 | 45137 | Endonuclease | 130 | Escherichia phage vB_EcoM_ECOO78 | YP_009600700.1 | 44.85 | 98 | 3.00E-31 |
| 66 | 45137 | 45505 | Single strand DNA binding protein | 122 | Escherichia phage Mangalitsa | YP_009850485.1 | 73.39 | 100 | 1.00E-47 |
| 67 | 45639 | 45839 | Hypothetical protein | 66 | Yersinia phage PYps16N | YP_010844674.1 | 87.88 | 100 | 9.00E-38 |
| 68 | 45839 | 46126 | Hypothetical protein | 95 | Escherichia phage vB_EcoM_Bp10 | YP_010844922.1 | 98.95 | 100 | 9.00E-61 |
| 69 | 46104 | 46334 | Hypothetical protein | 76 | Escherichia phage SKA49 | YP_010844756.1 | 97.14 | 92 | 4.00E-41 |
| 70 | 46348 | 46638 | Hypothetical protein | 96 | Escherichia phage ST32 | YP_009790674.1 | 96.88 | 100 | 1.00E-61 |
| 71 | 46702 | 46932 | Hypothetical protein | 76 | Escherichia phage ST32 | YP_009790673.1 | 63.16 | 100 | 7.00E-29 |
| 72 | 46929 | 47156 | Hypothetical protein | 75 | Escherichia phage flopper | YP_009855567.1 | 96 | 100 | 3.00E-47 |
| 73 | 47159 | 47341 | Hypothetical protein | 60 | Escherichia phage ST32 | YP_009790671.1 | 95 | 100 | 9.00E-35 |
| 74 | 47334 | 47594 | Hypothetical protein | 86 | Escherichia phage PO103-1 | YP_010844413.1 | 98.84 | 100 | 6.00E-56 |
| 75 | 47691 | 47864 | Hypothetical protein | 57 | Escherichia phage PO103-1 | YP_010844414.1 | 98.25 | 100 | 1.00E-33 |
| 76 | 47865 | 48029 | Hypothetical protein | 54 | Escherichia phage SKA49 | YP_010844750.1 | 96.3 | 100 | 7.00E-29 |
| 77 | 48026 | 48196 | Hypothetical protein | 56 | Escherichia phage PO103-1 | YP_010844416.1 | 85.71 | 100 | 6.00E-25 |
| 78 | 48196 | 48384 | Hypothetical protein | 62 | Escherichia phage vB_EcoM_Bp10 | YP_010844914.1 | 98.39 | 100 | 2.00E-36 |
| 79 | 48386 | 48688 | Hypothetical protein | 100 | Yersinia phage PYps16N | YP_010844666.1 | 90 | 100 | 2.00E-60 |
| 80 | 48630 | 48881 | Hypothetical protein | 83 | Escherichia phage MLP1 | YP_010844452.1 | 98.8 | 100 | 1.00E-52 |
| 81 | 48871 | 49011 | Hypothetical protein | 46 | Yersinia phage PYps16N | YP_010844664.1 | 73.08 | 57 | 0.031 |
| 82 | 49632 | 49826 | Hypothetical protein | 64 | No significant similarity found |  |  |  |  |
| 83 | 50081 | 50245 | Hypothetical protein | 54 | Escherichia phage MLP1 | YP_010844450.1 | 88.89 | 100 | 4.00E-27 |
| 84 | 50339 | 50539 | Hypothetical protein | 66 | Escherichia phage vB_EcoM_Bp10 | YP_010844910.1 | 98.48 | 100 | 7.00E-41 |
| 85 | 50589 | 52529 | RNA polymerase | 647 | Escherichia phage ST32 | YP_009790661.1 | 97.37 | 100 | 0 |

**Table S3** Summary of phage growth parameters of members in the family *Chaseviridae*

| **Phage name** | **Parental host ^a^** | **Genus** | **Tested temperature (*◦*C)** | **Adsorption period**  **(mpi) ^b^** | **Adsorption (%) ^C^** | **One-step growth assay** | | **NCBI accession number** | **Reference** |
| --- | --- | --- | --- | --- | --- | --- | --- | --- | --- |
|  |  |  |  |  |  | **Latent period (mpi)** | **Burst size**  **(particles per infected cell)** |  |  |
| Tiny | *E. coli* ATCC 25922 | *Carltongylesvirus* | 30 | 25 | 43 ± 6.24 | 70 | 58 ± 6 | PX572931 | This study |
| vB_EcoM-4HA13 | *E. coli* non-O157 STEC strain O111:NM | *Sabourvirus* | 25 | 2 | 57 | 90 | 55 ± 10 | NC_049466.2 | Lin *et al*. (1) |
| ST32 | *E. coli* STEC strain ST130 | *Carltongylesvirus* | 20 | No report | No report | 102 | 602 ± 159 | MF044458.2 | Liu *et al*. (2) |
|  |  |  | 30 | No report | No report | 54 | 64 ± 30 |  |  |
|  |  |  | 37 | No report | No report | 55 | 2 ± 0.1 |  |  |
| SKA49 | *E. coli* APEC strain QZJM25 | *Carltongylesvirus* | 37 | No report | No report | 35 | 56 ± 7 | NC_079180.1 | Sattar *et al.* (3) |
| PP101 | *Pectobacterium brasiliense* strain F152 (PB29) | *Suwonvirus* | 28 | 4-6 | >90 | 30–35 | 100-150 | KY087898 | Lukianova *et al*. (4) |

^a^ The phage replication cycle is dependent on the bacterial host strain; therefore, phage growth parameters vary with different hosts. The growth parameters of phage Tiny are shown for the parental host, *E. coli* ATCC 25922 (Fig. S1), while those for other strains are presented in Fig. 4.

^b^ The adsorption period represents the time required for phage attachment to bacterial cells prior to the onset of the equilibrium phase.

^c^ The percentage of adsorption was determined during the adsorption period by quantifying the reduction in free (unattached) phage, reflecting the proportion of virions adsorbed to bacterial cells.

^d^ The latent period and burst size values summarized in this table were compiled from different studies and may vary due to differences in experimental conditions, including MOI, culture temperature, host strain, and adsorption time.

**References**

1. Lin JT, Kirst S, Cucić S, Klem A, She Y-M, Kropinski AM, Anany H. 2022. Isolation, characterization, and genome analysis of a novel bacteriophage, *Escherichia* phage vB_EcoM-4HA13, representing a new phage genus in the novel phage family *Chaseviridae*. Viruses 14:2356. https://doi.org/10.3390/v14112356

2. Liu H, Geagea H, Rousseau GM, Labrie SJ, Tremblay DM, Liu X, Moineau S. 2018. Characterization of the *Escherichia coli* virulent myophage ST32. Viruses 10:616. https://doi.org/10.3390/v10110616

3. Sattar S, Bailie M, Yaqoob A, Khanum S, Fatima K, Altaf AURB, Ahmed I, Shah STA, Munawar J, Zehra QA, Daud S, Arshad A, Imdad K, Javed S, Tariq A, Bostan N, Altermann E. 2023. Characterization of two novel lytic bacteriophages having lysis potential against MDR avian pathogenic *Escherichia coli* strains of zoonotic potential. Sci Rep 13:10043. <https://doi.org/10.1038/s41598-023-37176-z>

4. Lukianova AA, Shneider MM, Evseev PV, Shpirt AM, Bugaeva EN, Kabanova AP, Obraztsova EA, Miroshnikov KK, Senchenkova SN, Shashkov AS, Toschakov SV, Knirel YA, Ignatov AN, Miroshnikov KA. 2020. Morphologically different *Pectobacterium brasiliense* bacteriophages PP99 and PP101: Deacetylation of O-polysaccharide by the tail spike protein of phage PP99 accompanies the infection. Front microbiol Volume 10 - 2019: <https://doi.org/10.3389/fmicb.2019.03147>
